# Chronic Periodontitis Is Associated with the Risk of Bipolar Disorder: A Population-Based Cohort Study

**DOI:** 10.3390/ijerph17103466

**Published:** 2020-05-15

**Authors:** Yung-Kai Huang, Yu-Hsun Wang, Yu-Chao Chang

**Affiliations:** 1 Department of Oral Hygiene, College of Dental Medicine, Kaohsiung Medical University, Kaohsiung 80708, Taiwan; ykhuang@kmu.edu.tw; 2 Department of Medical Research, Chung Shan Medical University Hospital, Taichung 40201, Taiwan; cshe731@csh.org.tw; 3 School of Dentistry, Chung Shan Medical University, Taichung 40201, Taiwan; 4 Department of Dentistry, Chung Shan Medical University Hospital, Taichung 40201, Taiwan

**Keywords:** bipolar disorder, chronic periodontitis, cohort study, Taiwan

## Abstract

Bipolar disorder (BD) is a psychiatric mood disturbance manifested by manic, hypomanic, or major depressive periods. Chronic inflammation was evidenced as an important etiologic factor of BD. Chronic periodontitis (CP) is an inflammatory disease triggered by bacterial products, leading to the destruction of periodontium. The relationship between BD and CP is of interest to investigate. Therefore, a nationwide population-based cohort study was used to investigate the risk of BD and CP exposure from 2001 to 2012. We identified 61,608 patients with CP from the Taiwanese National Health Insurance Research Database (NHIRD). The 123,216 controls were randomly captured and matched by age, sex, index year, and co-morbidities. The association between CP exposure and BD risk was examined by Cox proportional hazards regression models. In this study, 61,608 CP patients and 123,216 controls were followed up for 7.45 and 7.36 years, respectively. In total, 138 BD patients were identified in the CP cohort and 187 BD cases were found in the non-CP cohort. The incidence rate of BD was significantly higher in the CP cohort than in the non-CP cohort (adjusted HR: 1.46, 95% CI: 1.17–1.81) according to the multivariate Cox regression analysis. Females had a 1.47-fold increased risk (95% CI: 1.16–1.86) for BD compared to males. Taken together, CP may be associated with an increased risk of subsequent BD in Taiwan.

## 1. Introduction

Bipolar disorder (BD) is known as a manic-depressive illness related to substantial functional and cognitive deficits [1,2] and increased suicidal risk [3]. The symptoms of this mental disorder can manifest as manic, hypomanic, or major depressive episodes [4]. To date, the etiology of BD is not well known. Genetic influences, environmental factors, psychosocial variables, and neurological injury may play an important role in the development and course of BD [5,6,7]. Recently, cumulative evidence has revealed that BD was relevant to low-grade chronic inflammation, excessive oxidative stress, and immune reactions in the peripheral nervous system and the brain [8,9].

Chronic periodontitis (CP) is evoked by the periodontal pathogens and the products could induce an immune-inflammatory response in periodontium [10,11]. Clinical symptoms manifest as subsequent gingival bleeding, pocket formation, alveolar bone resorption, and even tooth loss. The destruction of periodontium, inflammatory and immunologic reactions may be related to the excessive production of reactive oxygen species [11,12]. In a review of the evidence, CP is also associated with many systemic diseases, such as cardiovascular diseases [13], neurodegenerative diseases [14,15], and immune-related diseases [16].

From the aforementioned descriptions, BD and CP evidently share common risk factors and co-morbidities. The pathogeneses of BD and CP might influence each other. Recently, one case-control study reported that BD was related to the increased risk of periodontitis [17]. However, there were no cohort design and large-scale population to address the relationship between CP and BD. In this study, we designed a population-based cohort study to investigate the risk of BD in patients with CP from the Taiwanese National Health Insurance Research Database (NHIRD).

## 2. Materials and Methods

### 2.1. Database

The Longitudinal Health Insurance Database 2010 (LHID2010), representing the entire NHIRD, was selected for this cohort study. The LHID2010 contained all of the original claim data of one million individuals randomly sampled from the registry for beneficiaries of the NHIRD in 2010 [18]. There were no significant differences in age and sex between the subjects in the LHID2010 and the NHIRD. The LHID2010 also contained demographic data, such as birthday, sex, clinical diagnoses, and health issues, as described previously [19,20]. The study protocol was authorized by the institutional review board of Chung Shan Medical University Hospital (CS2-15017).

### 2.2. Study Design and Population

**Figure 1:**
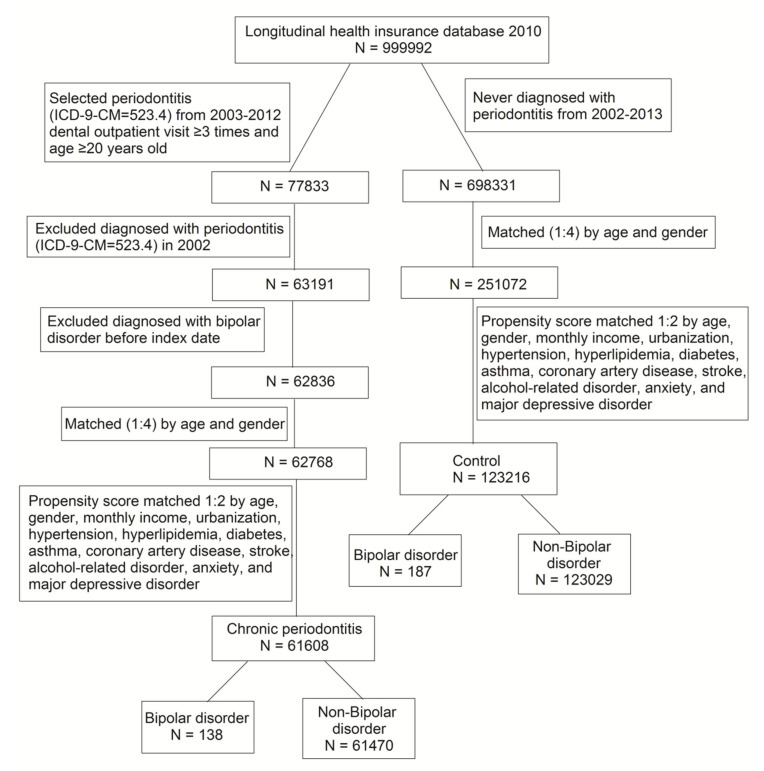
Procedures used for selection of cases from the Longitudinal Health Insurance Database 2010.

We identified CP cases based on International Classification of Diseases, 9th Revision, Clinical Modification (ICD-9-CM) code 523.4 in the LHID2010. The ambulatory patients for dental visits with diagnosed CP from 2003 to 2012 were captured as a CP cohort. Patients with the diagnosis of periodontitis before 2002 were excluded. The index date was recognized as the first diagnosis date of CP. To increase the validity of CP diagnoses, we recruited only the patients who had made at least three dental visits. In order to confirm the new-onset CP, the diagnosis of BP before the index date was excluded. The non-CP cohort was never diagnosed with periodontitis from 2002 to 2013. The 1:4 age and gender matched comparisons were selected to give the corresponding index date for the non-CP cohort. Furthermore, we performed the 1:2 propensity score matching by age, gender, monthly income, urbanization, hypertension, hyperlipidemia, diabetes, asthma, coronary artery disease, stroke, alcohol-related disorder, anxiety, and major depressive disorder. The propensity score was calculated by logistic regression that CP and non-CP were the binary outcome. By matching the propensity score, we could reduce the heterogeneity between the two groups. The study flow diagram of inclusion and exclusion criteria is shown in Figure 1.

### 2.3. Co-Morbidities

The potential baseline characteristics were determined for each patient by monthly income, urbanization, and comorbidities, including hypertension (401–405), hyperlipidemia (272.0–272.4), diabetes (250), asthma (493), coronary artery disease (410–414), stroke (430–438), alcohol-related disorder (291, 303, 305.0, 571.0, 571.1, 571.3, 790.3, and V11.3), anxiety (300.00, 300.01, 300.02, and 300.09), and major depressive disorder (296.2–3). These comorbidities were defined as diagnoses one year before the index date.

### 2.4. Outcome Measurement

All study patients were followed from the index date until the first diagnosis of BD, date of death, withdrawal from the NHI program, or December 31, 2013 (whichever came first). Patients with BD were identified by ICD-9-CM codes 296.0X, 296.1X, 296.4X, 296.5X, 296.6X, 296.7X, 296.80, or 296.89. To ensure the validity of BD diagnoses, only the drugs (lithium, valproate, carbamazepine, lamotrigine, aripiprazole, olanzapine, quetiapine, risperidone, and ziprasidone) approved by the Taiwan Food and Drug Administration for treating one or more phases of BD were captured. We only included patients who took the aforementioned drugs at least one month. In this study, the mean follow-up duration of BD was 7.45 years and 7.36 years in the CP and non-CP cohort, respectively. The mean time to BD was 4.12 and 4.56 years in CP and non-CP cohort, respectively.

### 2.5. Statistical Analysis

Data were analyzed using SPSS version 18 (SPSS, Chicago, IL, USA). Demographic characteristics for the CP and non-CP groups were analyzed using the Student’s *t* test and the Chi-square test. The Kaplan–Meier curve was used to perform the cumulative incidence of the CP and non-CP cohort and the Log-rank test was to examine the significance. Hazard ratios were estimated by Cox proportional hazard models. Probability levels of <0.05 were considered significant.

## 3. Results

As shown in Table 1, we evaluated 61,608 patients in the CP-cohort and 123,216 subjects in the comparison cohort, respectively. The demographic characteristics and selected co-morbidities were similar between the two groups.

As shown in Table 2, the number of newly-diagnosed BD patients was 138 in the CP cohort, and 187 individuals in the comparison cohort. The incidence density rate of BD in the comparison cohort was 0.2 per 1000 person-years, but the incidence density rate was approximately 1.5-fold higher in the CP cohort than in the comparison cohort. CP patients revealed a 1.46-fold increased risk of BD compared with non-CP patients (95% CI: 1.22–1.91), after adjustment of sex, age, monthly income, urbanization, and co-morbidities.

The age-specific adjusted hazard ratio (HR) HR of BD decreased 0.77-fold in those aged ≥65 years, compared with the group aged 20–64 (Table 2). The female group had higher rates of BD than the male group (adjusted HR: 1.47; 95% CI: 1.16–1.86). The higher monthly income group had a lower risk for BD (adjusted HR: 0.50; 95% CI: 0.37–0.69). In addition, patients with an alcohol-related disorder, anxiety, or major depressive disorder revealed a significant risk for BD (*p* < 0.001). However, there was no significant risk for BD with urbanization, hypertension, hyperlipidemia, diabetes, asthma, and coronary artery disease in the adjusted model.

Table 3 showed subgroup analysis of hazard ratios of BD development. The HR of BD increased 1.37-fold in CP patients aged 20-64, compared with non-CP patients within the same age group. The CP patients who were above 65 years old showed a 2.40-fold increased BD risk compared with non-CP patients who were above 65 years old. Compared with non-CP patients, the male CP patients had a 2.03-fold increased BD risk (95% CI: 1.40–2.94, *p* < 0.001).

**Figure 2:**
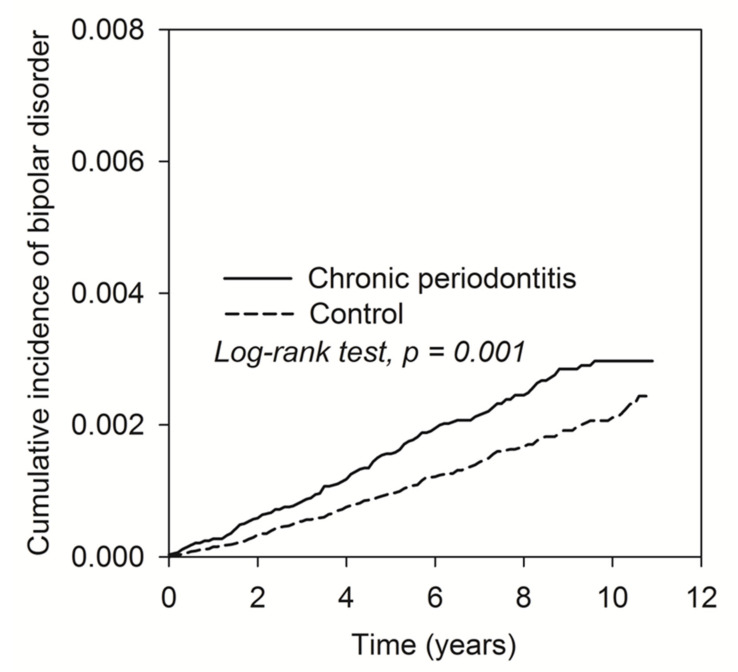
The Kaplan–Meier plot for the cumulative incidence of BD in patients with CP and control subjects.

Figure 2 shows the cumulative curve of the BD incidence. The results demonstrated that the curve of CP patients was significantly higher than the curve of control subjects (log rank test, *p* = 0.001).

## 4. Discussion

To the best of our knowledge, we first reported a higher risk of BD in patients with CP exposure than those who never received a diagnosis of CP from the nationwide population-based cohort study. Similar results were reported by Cunha et al. [17] who implied the positive relationship between BD and CP by a case-control study. However, limited samples, the use of only one hospital, cross sectional design, and lacking confounders could create a bias. The strength of this study was that it was conducted by a cohort design using the whole Taiwanese population. Some confounding factors, including income, urbanization, and medical comorbidities, were also adjusted. Diagnostic and prescription data were retrieved from a registry database that collected all medical claims, so the possibility of recall bias could be minimized. Moreover, the cohort study design could demonstrate a more causal relationship than a cross sectional case-control study design.

Our study revealed potential risk factors related to the subsequent occurrence of BD, such as sex, financial income, and comorbidities. The sex difference in this study might be partly explained by the fact that female BD patients sought treatment more often than males did [21]. In Taiwanese NHIRD, monthly income represents socioeconomic status. BD is obviously associated with lower socioeconomic outcomes [22]. Alcohol use and BD were found to share certain common genetic characteristics and biochemical findings [23]. Anxiety has usually been reported as comorbid with BD [24]. Patients who suffered from major depression may have unrecognized BD [25]. In addition, depressive symptoms were present within episodes during the course of BD [26]. Taken together, these confounding factors were associated with the subsequent occurrence of BD.

The reason why CP patients had a higher risk for BD may be explained by the inflammation induced by periodontal pathogens. CP is a chronic inflammatory disease caused by periodontal pathogens, triggering host inflammatory-immunologic responses. Patients with BD exhibited significantly higher amounts of periodontal pathogens *A. actinomycetemcomitans* and *P. gingivalis* [17]. Periodontal pathogens and virulent products might directly invade the brain via the circulatory system, and then activate microglia and the immune cells, resulting in neuro-inflammation [27].

The other possibility explaining why CP may cause BD is suggested by the presumption that the upregulation of pro-inflammatory cytokines within the central nervous system is associated with chronic systemic inflammation, which is triggered by CP. Elevation of pro-inflammatory cytokines IL-2, IL-4, and IL-6 has been reported during manic periods, whereas IL-6 is highly expressed during depressive periods [28]. Pro-inflammatory cytokines act as key inflammatory mediators in the development of CP. Moreover, reports have revealed that IL-6 is significantly higher in CP patients than healthy individuals [29,30]. However, this speculation still needs further investigation.

Some potential limitations must be mentioned. Firstly, BD is diagnosis by ICD-9 codes, so only those who pay a visit and admit their diagnosis are identified in the NHIRD. BD patients always have poor adherence to their medications. Therefore, BD prevalence may be underestimated in both study groups. However, the diagnosis of BP in the NHIRD is verified by board-certified psychiatrists. The collected data, based on prescriptions of psychotropic agents for these patients, could ensure that the BD diagnoses in the database were reliable. Secondly, CP diagnosis was also based on the ICD-9 code in this study. However, the terminology of CP is not appropriate after the new classification scheme for periodontal and peri-implant diseases and conditions in 2018 [31]. The ICD system could not meet the criteria of new classifications by the American Academy of Periodontology and the European Federation of Periodontology. This discrepancy needs to be noted, for the interpretation of the risk of BD in patients with periodontal diseases. Thirdly, this is a registry-based study. Therefore, patients with undiagnosed periodontitis might have been captured in the control group. Fourthly, several potential risk factors were also unavailable in the database, such as obesity, smoking, frequency of brushing teeth, stress, and family medical history. However, we used propensity score matching that could reduce the selection bias and avoid the confounding. Finally, due to the observational design of this study, further prospective studies are required to clarify the relationship between CP exposure and the increased risk of BD.

## 5. Conclusions

The findings of this population-based cohort study suggest that CP exposure may increase the risk of subsequent BD. Additional studies are warranted to clarify the causal relationship between the two diseases.

## Figures and Tables

**Table 1:** Characteristics of patients with chronic periodontitis and matched cohort.

	Chronic Periodontitis (*N* = 61,608)		Control (*N* = 123,216)		
	*n*	%	*n*	%	*p*-Value
Age					0.533
20–64	55,240	89.7	110,591	89.8	
≥65	6368	10.3	12,626	10.2	
Mean ± SD	44.77 ± 14.75		44.8 ± 14.69		0.700
Gender					0.344
Male	29,080	47.2	57,873	47.0	
Female	32,528	52.8	65,343	53.0	
Monthly income					0.731
<NT $20,000	26,114	42.4	52,049	42.2	
NT $20,000-NT $40,000	19,223	31.2	38,663	31.4	
>NT $40,000	16,271	26.4	32,504	26.4	
Urbanization					0.789
Urban	41,880	68.0	83,838	68.0	
Suburban	16,265	26.4	32,548	26.4	
Rural	3463	5.6	6830	5.5	
Hypertension	8314	13.5	16,782	13.6	0.460
Hyperlipidemia	4302	7.0	8566	7.0	0.806
Diabetes	3754	6.1	7569	6.1	0.676
Asthma	1015	1.6	1965	1.6	0.396
Coronary artery disease	2507	4.1	5000	4.1	0.907
Stroke	1191	1.9	2346	1.9	0.666
Alcohol-related disorder	126	0.2	227	0.2	0.346
Anxiety	2115	3.4	4164	3.4	0.549
Major depressive disorder	248	0.4	463	0.4	0.381

The Student’s *t* test and Chi-squared test were used to test the difference of continuous and categorical variables, respectively.

**Table 2:** Risk factor analysis of bipolar disorder development.

	No. of Event	Observed Person-Years	ID	Crude HR	95% C.I.	*p*-Value	Adjusted HR ^†^	95% C.I.	*p*-Value
Chronic periodontitis									
No	187	907,027	0.2	1			1		
Yes	138	459,080	0.3	1.46	1.17–1.82	<0.001	1.46	1.17–1.81	<0.001
Age									
20–64	289	1,234,117	0.2	1			1		
≥65	36	131,990	0.3	1.17	0.83–1.65	0.374	0.77	0.52–1.14	0.188
Gender									
Male	112	639,044	0.2	1			1		
Female	213	727,063	0.3	1.67	1.33–2.10	<0.001	1.47	1.16–1.86	0.001
Monthly income									
<NT $20,000	184	577,755	0.3	1			1		
NT $20,000–NT $40,000	88	425,043	0.2	0.65	0.50–0.84	<0.001	0.67	0.52–0.86	0.002
>NT $40,000	53	363,309	0.1	0.46	0.34–0.62	<0.001	0.50	0.37–0.69	<0.001
Urbanization									
Urban	222	927,506	0.2	1			1		
Suburban	86	362,896	0.2	0.99	0.77–1.27	0.935	1.00	0.78–1.28	0.991
Rural	17	75,705	0.2	0.94	0.57–1.54	0.800	0.95	0.58–1.55	0.827
Hypertension	53	173,558	0.3	1.35	1.00–1.81	0.048	0.89	0.63–1.27	0.536
Hyperlipidemia	31	86,356	0.4	1.58	1.09–2.28	0.016	1.16	0.77–1.77	0.476
Diabetes	28	77,185	0.4	1.58	1.08–2.34	0.020	1.26	0.82–1.95	0.289
Asthma	8	21,439	0.4	1.59	0.79–3.20	0.197	1.09	0.54–2.22	0.810
Coronary artery disease	24	52,610	0.5	2.00	1.32–3.03	0.001	1.48	0.94–2.34	0.093
Stroke	10	23,871	0.4	1.80	0.96–3.37	0.068	1.27	0.66–2.46	0.475
Alcohol–related disorder	8	2369	3.4	14.62	7.25–29.5	<0.001	10.31	5.04–21.08	<0.001
Anxiety	55	43,852	1.3	6.17	4.62–8.25	<0.001	3.76	2.72–5.21	<0.001
Major depressive disorder	32	4882	6.6	30.60	21.24–44.08	<0.001	15.10	10.11–22.56	<0.001

ID: Incidence density, per 1000 person-years. HR: Hazard ratio. ^†^ Adjusted for age, gender, monthly income, urbanization, hypertension, hyperlipidemia, diabetes, asthma, coronary artery disease, stroke, alcohol-related disorder, anxiety, and major depressive disorder.

**Table 3:** Subgroup analysis of Hazard ratios of bipolar disorder.

	Chronic Periodontitis		Control		HR	95% C.I.	
	N	No. of Event	N	No. of Event			*p*-Value
Age							
20–64	55,240	118	110,590	171	1.37	1.08–1.73	0.009
≥65	6368	20	12,626	16	2.40	1.24–4.63	0.009
							*p* for interaction = 0.119
Gender							
Male	29,080	57	57,873	55	2.03	1.40–2.94	<0.001
Female	32,528	81	65,343	132	1.22	0.93–1.61	0.159
							*p* for interaction = 0.031
